# Trends in Food Group Intake According to Body Size among Young Japanese Women: The 2001–2019 National Health and Nutrition Survey

**DOI:** 10.3390/nu14194078

**Published:** 2022-09-30

**Authors:** Mai Matsumoto, Ryoko Tajima, Aya Fujiwara, Xiaoyi Yuan, Emiko Okada, Hidemi Takimoto

**Affiliations:** 1 Department of Nutritional Epidemiology and Shokuiku, National Institutes of Biomedical Innovation, Health and Nutrition, 1-23-1 Toyama, Shinjuku-ku, Tokyo 162-8636, Japan; 2 Department of Social and Preventive Epidemiology, School of Public Health, University of Tokyo, Bunkyo-ku, Tokyo 113-8654, Japan; 3 Department of Epidemiology and Prevention, Center for Clinical Sciences, National Center for Global Health and Medicine, Tokyo 162-8655, Japan

**Keywords:** food groups, Japan, NHNS, trend, young women

## Abstract

Unlike in many industrialised countries, the high proportion of young women who are underweight in Japan has been a long-term problem. We evaluated trends in food group intake according to body size among young Japanese women using data from the National Health and Nutrition Survey 2001–2019. Overall, 13,771 Japanese women aged 20–39 years were included. A 1-day household-based dietary record was used to estimate food intake. Foods were classified into 34 groups based on the Standard Tables of Food Composition in Japan. The trend of food group intake was analysed using the Joinpoint Regression Program. The proportion of young women who were underweight was consistently around 20%, while obesity among young women increased between 2001 (10%) and 2019 (13%). A decreased trend in fish and shellfish and seaweed intake and an increased trend in meat and soft drink intake were observed among young women. Decreased trends in the intake of fruit and dairy products were observed in young women who were not obese. An increased trend in the intake of confectionaries was observed in young women who were obese. This study suggests that the types of unhealthy eating habits may differ according to body size among young Japanese women.

## 1. Introduction

Obesity is associated with the development of chronic diseases such as hypertension, type 2 diabetes, cardiovascular disease, and hypercholesterolaemia [1], which continues to increase among many age groups and is considered a major public health problem worldwide [2]. In Japan, the prevalence of obesity among adults (men, 33%; women, 22%) is lower than in other countries (men, 39%; women, 40%) [2,3], and is particularly low among young Japanese women (around 10%) in their 20s and 30s [3]. Meanwhile, the proportion of Japanese women who are underweight has been reported to be high (around 20%) for over 40 years [3,4]. Most other countries have shown a decline in women who are underweight; however, it has been reported that the proportion is increasing in China [5] and Poland [6]. Young women who are underweight also have raised susceptibility to several negative health conditions, such as low muscle mass [7], low bone density [8], and unfavourable pregnancy outcomes, such as having low birth weight infants [9]. Therefore, it is necessary to continuously monitor the weight status of the population and generate appropriate public policies targeting both obesity and those who are underweight, in order to increase the proportion of adequate-weight individuals.

Several studies have reported that body mass index (BMI) is related to the intake of certain foods. For example, higher fruit, vegetable, and nut intake were related to lower BMI, while higher intake of meat, confectionaries, and soft drinks may be associated with a higher BMI [10,11,12,13]. A higher intake of mushrooms, seafood, potatoes, seaweed, and soy products has also been reported to be associated with a lower risk of obesity (BMI ≥ 25 kg/m^2^) [14]. In addition, the difference in BMI among young women was reported to be associated with not only the type but also the number of consumed foods [6]. Thus, the assessment of the relationship between body size and food group intake trends among young women is crucial for health promotion. Food group intake trends have been reported to change over time, and have become more Westernised in East Asian countries, including Japan [15,16]. To the best of the authors’ knowledge, no studies have evaluated trends in food group intake according to body size. Therefore, the present study aimed to examine the trends regarding food group intake according to body size among young Japanese women using data from the National Nutrition Survey (NNS) and National Health and Nutrition Survey (NHNS) from 2001 to 2019.

## 2. Materials and Methods

### 2.1. Study Design and Participants

The NNS (until 2002) and NHNS (renamed since 2003) in Japan are nationally representative cross-sectional annual surveys conducted by local public health centres under the supervision of the Ministry of Health, Labour, and Welfare [17]. This study evaluated data from the NNS (2001, 2002) and NHNS (2003–2019), because the NNS used to assign specific food codes to food items until 2000. However, the food codes used by the NNS and NHNS have been the same as the food codes in the Standard Tables of Food Composition since 2001. The details of the survey design have been described elsewhere [17,18]. In brief, the surveys were conducted in November, except for the 2012 (conducted from 25 October to 7 December) and 2016 surveys (conducted from 1 October to 30 November). The NNS and NHNS consisted of a physical examination, dietary survey, and lifestyle questionnaire. Participants were household members aged ≥1 year residing in 300-unit blocks (about 5700 households and 15,000 people) that were randomly selected from the unit blocks of the Comprehensive Survey of Living Conditions each year, excluding 2012 and 2016 when an expanding survey was conducted. In 2012 and 2016, 475 units were randomly stratified using a single-stage cluster sample design from the Population Census unit, and all household members aged ≥1 year in the selected area (approximately 23,750 households and 61,000 individuals) were designated. The following areas in the relevant year were not surveyed because of natural disasters: Iwate, Miyagi, and Fukushima prefectures due to the Great East Japan Earthquake in 2011 [19]; Kumamoto due to the Kumamoto earthquake and Typhoon No. 10 and some areas of Tottori prefectures due to the Tottori Prefecture earthquake in 2016 [20], as well as some areas of Nagano prefecture due to Typhoon Hagibis in 2019 [3]. Household response rates for each year in the NNS and NHNS ranged from 44·4% in 2016 to 83.2% in 2003 [17]. This study included 20,654 women aged 20–39 years who participated in a dietary survey. We excluded lactating or pregnant women who may have changed their usual dietary habits (*n* = 1992) [21] and those with missing data, such as body height and/or body weight (*n* = 4357), and step counts (*n* = 1650), and there were some participants without several variables. Thus, the final participants in the present study were 13,771 young Japanese women aged 20–39 years.

The NNS and NHNS were conducted in accordance with the guidelines laid down in the Declaration of Helsinki. As the survey was conducted according to the Health Promotion Act [22], all participants provided informed consent to the local government. Permission to use the data of the NNS and NHNS was obtained from the Ministry of Health, Labour, and Welfare, and only anonymised information was available for the present study. Approval from the Institutional Review Board was not required.

### 2.2. Dietary Survey

The dietary survey was conducted using a single day semi-weighed household dietary record, with the exemption of Sundays and public holidays [17]. Trained fieldworkers, mainly registered dieticians, visited each household to explain how to complete the dietary record. The main record-keepers, members who are usually responsible for preparing meals in the household, weighed all foods and beverages consumed in the household, food waste, and leftovers. For shared dishes in the household, the approximate proportions of foods were assigned to each household member to estimate an individual’s food intake. If weighing was not possible because of dining out, the main record-keepers questioned the family member regarding portion size and amount of food consumed, as well as leftovers. This information was subsequently recorded.

After a dietary recording day, trained fieldworkers visited each household and checked the dietary record. For foods and beverages that were not weighed, the trained fieldworkers converted the portion sizes or amounts of foods, and recorded them as food weights. Each food item was then coded according to the NNS and NHNS food code lists based on the Standard Tables of Food Composition in Japan [23,24,25,26]. The trained fieldworkers finally inputted the collected dietary intake data using software specifically developed for the NNS and NHNS. The data were compiled at the central office [17].

Energy and macronutrients were calculated based on the Standard Tables of Food Composition in Japan 2000 edition (data from 2001 to 2006) [23], 2005 edition [24] (data from 2007 to 2010), 2010 edition [25] (data from 2011 to 2017), and 2015 edition [26] (data from 2018 to 2019). Additionally, based on the Standard Tables of Food Composition in Japan [23,24,25,26], we classified foods into 34 groups (Supplementary Table S1: Definition of food groups in this study). Food group intake was energy-adjusted using the density method (amount of food group per 1000 kcal).

A previous validation study comparing household-based dietary records and self-reported dietary records among young women has shown that the house-hold based method may underestimate energy, protein, fat, and carbohydrate by 6.2, 5.7, 6.7, and 6.3 percent, respectively [27].

### 2.3. Physical Assessments

Body height (to the nearest 0.1 cm) and weight (to the nearest 0.1 kg) were measured by trained field workers in accordance with the NNS and NHNS standard manual until 2005. Additionally, approximately 85% of the participants have been measured by trained field workers in accordance with the NHNS standard manual since 2006 [19], and the height and weights were measured by other family members or self-reported for the remainder of the participants. Body mass index (BMI) was calculated by dividing weight (kg) by height squared (m^2^). Participants were classified into three groups according to BMI defined by the Japan Society for the Study of Obesity (<18.5 kg/m^2^ as Underweight, 18.5–24.9 kg/m^2^ as Normal and ≥25.0 kg/m^2^ as Obese) [28]. Participants also wore a pedometer (AS-200, YAMASA, Tokyo, Japan) and measured their step counts on a single day.

### 2.4. Statistical Analysis

According to the survey years, mean values and standard errors for age, BMI, step counts, and intake of energy, macronutrients, and food groups were calculated. For the data from the 2012 and 2016 surveys, the sampling weight of participants in each prefecture was calculated by dividing the total number of households during the past three years in each prefecture by that in 2012 and 2016, respectively. This is because the cluster sampling method used for the extraction of participants in 2012 and 2016 was different from that used in other years [20,29]. Trend analyses were conducted using the Joinpoint Regression Program (Joinpoint Regression software, version 4.9.0.0; National Cancer Institute, Bethesda, MD, USA [https://www.surveillance.cancer.gov/joinpoint], accessed on 2 April 2021). Joinpoint regression analysis uses statistical criteria to determine the minimum number of linear segments required to describe a trend and the annual percentage change (APC) in each segment. The Monte Carlo permutation method was used to determine whether a change in the trend was statistically significant [30]. Other calculations and statistical analyses were conducted using SAS statistical software (version 9.4; SAS Institute Inc., Cary, NC, USA). Statistical significance was set at a two-tailed *p*-value of <0.05.

## 3. Results

**Figure 1:**
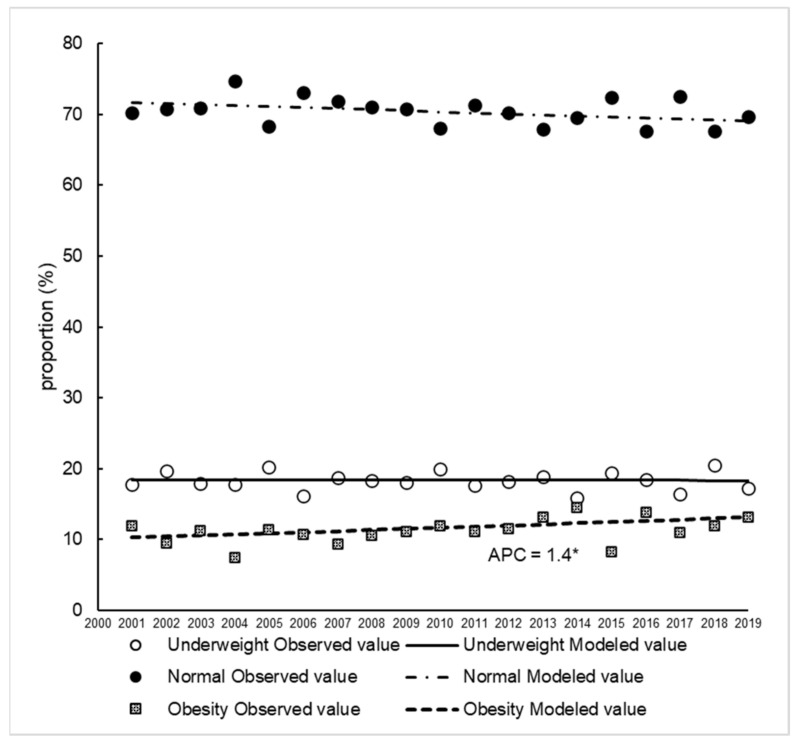
Trends in the proportion of young women classified as underweight, normal and obese between 2001 and 2019. Trends in the proportion of young women were analysed using Joinpoint regression analysis (*p* < 0.05). APC, annual percentage change. * *p* < 0.05.

The proportion of young women classified as underweight, normal, and obese from 2001 to 2019 are shown in Figure 1. There was no change in the proportion of young women who were underweight, which was consistently around 20%. The proportion of young women who were obese increased from approximately 10% in 2001 to approximately 13% in 2019 (APC = 1.4%).

**Figure 2:**
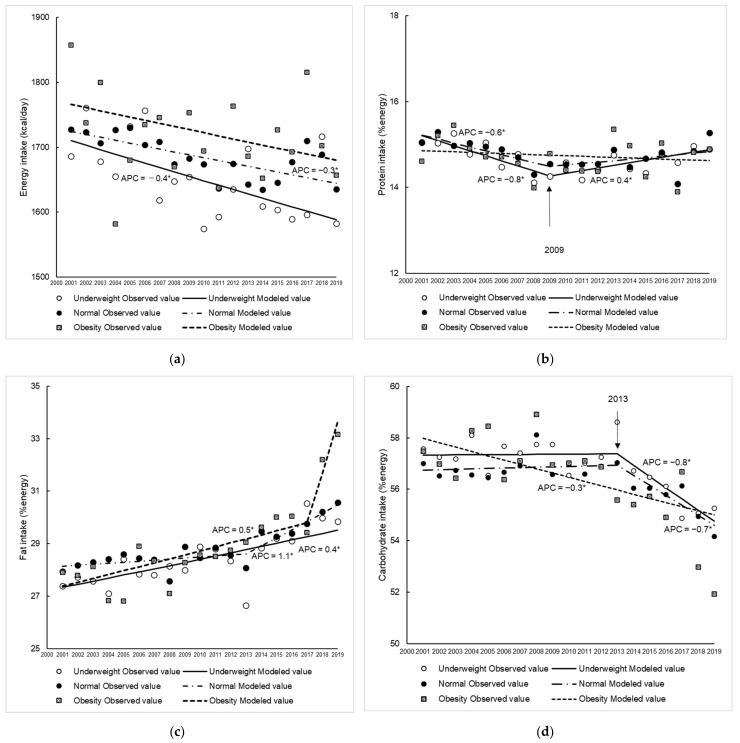
Trends in energy and macronutrient intake among young women classified as underweight, normal and obese between 2001 and 2019. Energy (**a**), protein (**b**), fats (**c**), carbohydrates (**d**). Trends in energy and macronutrient intake were analysed using Joinpoint regression analysis (*p* < 0.05). APC, annual percentage change. * *p* < 0.05.

The characteristics of the participants in the present study are shown in Table 1. For step counts, those in the underweight and normal groups decreased over time, but those in the obese group did not change. Trends in energy and macronutrient intake among young women according to BMI are shown in Figure 2a–d.

Energy intake in the underweight and normal groups decreased over time (annual decreases: 0.4% in the underweight group and 0.3% in the normal group). For protein intake, a decreasing trend was observed in the underweight and normal groups from 2001 to 2009. Thereafter, an increasing trend was only shown as significant in the underweight group, despite a similar trend being observed in the normal group. Fat intake increased in all groups, although the annual rate and duration of the increase varied (APC: 0.4% in the underweight group, 1.1% between 2013 and 2019 in the normal group, and 0.5% between 2001 and 2017 in the obese group). Carbohydrate intake decreased over time in the obese group (APC = −0.3%), whereas it remained unchanged until 2013 and then decreased at a greater rate in the underweight and normal groups (APC = −0.8% and −0.7%, respectively) than in the obese group.

**Figure 3:**
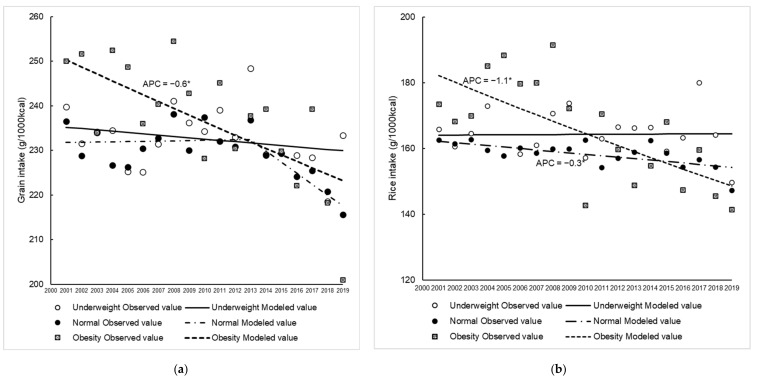

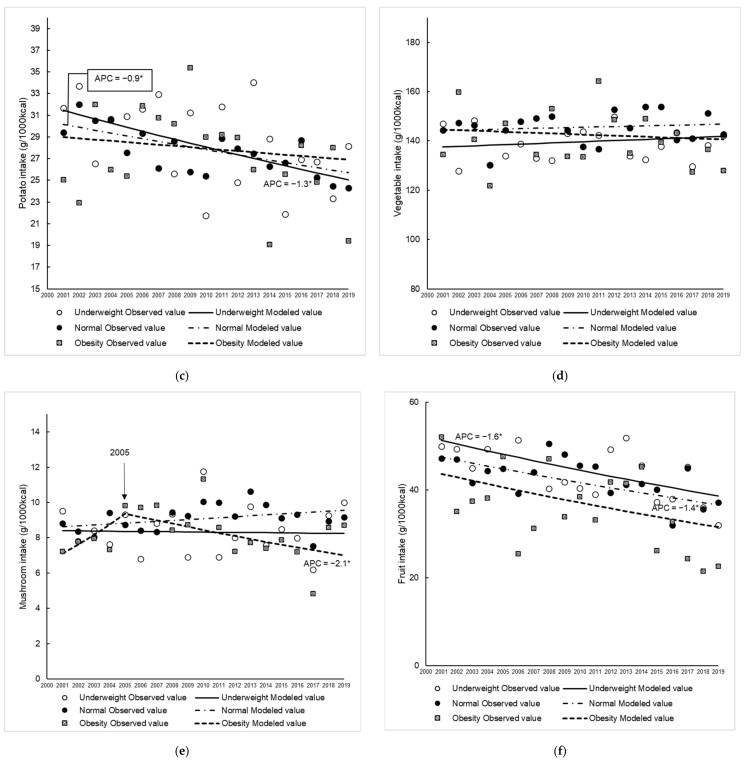

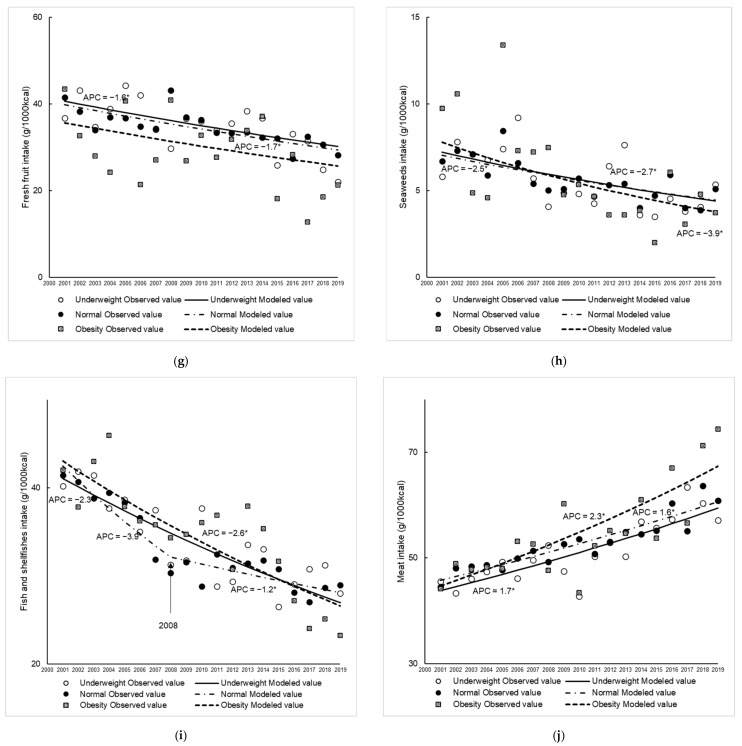

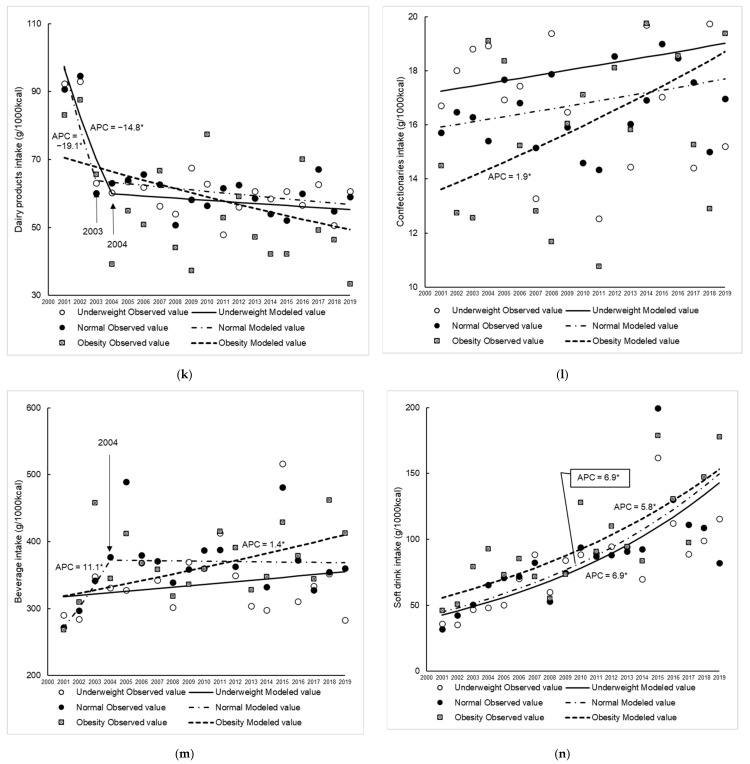
Trends in food group intake among young women classified as underweight, normal and obese between 2001 and 2019. Grains (**a**), rice (**b**), potatoes (**c**), vegetables (**d**), mushrooms (**e**), fruits (**f**), fresh fruits (**g**), seaweed (**h**), fish and shellfish (**i**), meat (**j**), dairy (**k**), confectionaries (**l**), beverages (**m**), soft drinks (**n**). Trends in food group intake were analysed using Joinpoint regression analysis (*p* < 0.05). APC, annual percentage change. * *p* < 0.05.

Figure 3a–n and Supplementary Table S2 show the trends in food group intakes between 2001 and 2019. In all groups, seaweed, fish, and shellfish intake decreased over time, whereas meat intake increased significantly in all groups. Grain intake in the obese group (APC = −0.6%) and rice intake in the normal (APC = −0.3%) and obese (APC = −1.1%) groups decreased over time. In the underweight and normal groups, the trend in potato intake also showed significant decreases with APC values of −1.3% and 0.9%, respectively, and a similar trend was observed for fruit intake (APC; underweight: −1.6%, normal: −1.4%), especially fresh fruit intake (APC; underweight: −1.6%, normal: −1.7%). Additionally, dairy product intake decreased sharply in the underweight group until 2004 and in the normal group until 2003 and did not change thereafter. In the obese group, an increase in confectionary intake was observed (APC = 1.9%). For beverage intake, an increasing trend was observed in the normal group between 2001 and 2004 (APC = 11.1%) and in the obese group between 2001 and 2019 (APC = 1.4%). Moreover, the trend in soft drink intake increased significantly in all groups (APC of 6.9% in the underweight group, 6.9% in the normal group, and 5.8% in the obese group).

## 4. Discussion

This study evaluated the trends in food group intake based on data from the 2001 to 2002 NNS and from 2003 to 2019 NHNS. Monitoring food group intakes over time in a representative sample of the Japanese population is one of the greatest strengths of the NNS and NHNS data. To our knowledge, this is the first study to evaluate the trends in food group intake among young women according to body size. We found decreasing trends in fish, shellfish and seaweed intake and increasing trends in meat and soft drink intake among young women. Additionally, decreased fruit and dairy product intake was observed in young women who were not obese, while increased confectionary intake was observed in young women who were obese.

The proportion of young Japanese women who were underweight was reported to have increased until 2000 [4], but the present results show that there has been no change in 20 years since 2000. However, it remains a problem, with as many as 1/5 of all young Japanese women still being underweight. On the other hand, the proportion of young women who are obese continues to increase, although not as much as in other foreign countries [2,31]. This suggests that the double burden of obesity and underweight individuals may start to develop among young women in Japan.

The number of step counts decreased over time in women who were underweight and normal weight, with a higher rate of decrease in women who were underweight. Energy intake also decreased in both groups. The mean BMI of participants in the normal group ranged between 20 and 21 kg/m^2^, which was close to the standard for thinness; 18.5 kg/m^2^. Young women who are underweight have been reported to have less muscle mass, less physical activity, and lower energy intake than those with an ideal weight [32,33]. It has been reported that young women are more likely to under-report their dietary intake [34] thus, the results of the present study should be interpreted with caution. However, it is possible that lower muscle mass and physical inactivity are associated with lower energy intake in underweight young women; therefore, it may be necessary to take these factors into account when considering dietary intake in the future.

In all groups, a decreasing trend was observed in the intake of fish, shellfish, and seaweed, which are commonly consumed in dietary patterns that are associated with a low risk of obesity [14]. An increasing trend in the intake of meat and soft drinks was also observed, which is reported to be positively associated with BMI [11,12,14]. This has resulted in the amount of meat intake being more than double that of fish intake over the last 20 years, which may contribute to the increased proportion of protein and fat intake. The trends in the intake of these food groups are also consistent with the results of the dietary patterns of Japanese adults in a previous study, which reported that dietary patterns among the Japanese population are westernised indicating a decrease in the “plant foods and fish” pattern and an increase in the “animal foods and oil” pattern [15]. It is highly possible that the diversification of the Japanese diet is one of the most significant factors contributing to the change in food group intake trends. Furthermore, it has been reported that the diet of young women may change as they move from living with family to living alone [35]. An increase in the number of one-person households has been reported in Japan [36], and it is possible that these factors may have influenced changes in dietary intake. Furthermore, young adults have recently been using social media as a source of food information more than other generations [3]. Given that social media has made great strides in the last decade [37], it is possible that these factors have had a significant impact on young adults’ dietary habits. With the changing social environment, future monitoring of dietary habits among young women should continue.

Contrary to the findings in the United States (U.S.), soft drink consumption per 1000 kcal in Japan is increasing rather than decreasing [38]. The inconsistency of the trends between Japan and the U.S. may be due to different policy measures made by the government and the public. Unlike in Japan, overweight individuals and obesity in the U.S. have long been regarded as public health concerns [39]. As a triggering factor for weight gain, soft drink consumption has been a target of social marketing (for example, expanding media coverage) and policymaking (for example, improving school meal programs) in public health to reduce its intake. Although obesity in Japan has not yet been as prevalent as in the U.S. (Approximately 22% (BMI ≥ 25 kg/m^2^) and 4% (BMI ≥ 30 kg/m^2^) in Japanese women versus around 40% (BMI ≥ 30 kg/m^2^) in US women) [3,40], the increased consumption of soft drinks may contribute to an increase in obesity in the future. As a previous study suggests, a woman’s dietary pattern may be unchanged before and after pregnancy [41]. The unfavourable changes in dietary intakes of Japanese young women, together with their decreased physical activity, may pose a risk to the health of future generations. Raising awareness regarding the current situation, as well as providing affordable and accessible healthy foods to the young generations may be necessary.

Although all young women had unhealthy eating behaviors, under- and normal weight women tended to consume fewer healthy foods, while obese women were likely to consume more unhealthy foods. This may suggest a need to address eating behavior according to body size. For example, fruit and dairy intake decreased over time in the under- and normal weight groups. Fruit and dairy products are high in vitamins and minerals [26] and have been reported to be associated with adequate nutrient intake [42,43]. Reports on the trend in fruit intake in other countries have been inconsistent [44,45,46]. Fruit intake increased over time in the U.S. [44], whereas a decreasing trend was observed in Hong Kong [45] and Canada [46]. Although the reason is unknown, a possible explanation for the increase in the U.S. was the success of a campaign highlighting the benefits of fruits [44]. In order to overcome the current situation in which the proportion of women who are underweight still accounts for 20% of the population, it is necessary to not only improve their weight status, but also promote a balanced diet by encouraging the consumption of fruit and dairy products to ensure future health for underweight (and normal weight) women. Meanwhile, in the obese group, an increase in confectionary intake was observed, which has been reported to be a predictor of unhealthy status, such as weight gain [10]. Additionally, grain intake decreased in the obese group. This may be one of the factors explaining the decrease in carbohydrate intake in the obese group in the present study. Among young women, the higher their BMI, the more likely they are to be on a diet, and consciously eat less than they would like to [47,48]. It has also been suggested that the desire to be thin in young Japanese women is related to less grain consumption, which may be due to the influence of carbohydrate-reducing diets in the media in recent years [49]. In the future, it may be necessary to consider dietary intake in the context of the food environment, including the influence of the media.

This study had some limitations. First, although participating households were randomly selected from a nationally representative sample in Japan, the individual-level response rate of household members is unknown. These factors may be biased in the estimation of the mean intake of foods. Second, it has been reported that the desire to be thin may significantly influence dietary intake among young women [48,49], but the willingness to be thin was not evaluated in the present study. It cannot be denied that this may have influenced the results. In the future, it will be necessary to consider dietary intake by taking into account the desire for thinness. Third, dietary assessment was mainly conducted in one day in November (including October and December in 2012 and 2016), excluding Sundays and public holidays. Therefore, dietary assessment could not take day-to-day variations into account for assessing the usual intake of food groups. Seasonal variations were not considered. The results should be interpreted with caution, since it has been reported that the intake of foods such as fruits, vegetables, and potatoes varies with regard to the season [50]. Fourth, the NHNS did not examine the disease status, and therefore young women who are underweight or obese due to any disease, including the side effects of medication, were not excluded. Fifth, approximately 15% of the participants reported their height and weight as measured at home or self-reported. These individuals were less likely to be obese than those who had been measured. This is because women reported that they were more likely to under-report their weight [51]. Therefore, the prevalence of obesity in the present study may have been underestimated.

## 5. Conclusions

This study identified a decrease trend in fish, shellfish and seaweed intake and an increase in meat and soft drink intake among young Japanese women. In addition, young Japanese women who were underweight or had a normal weight had a decreased intake of healthy foods, such as fruit and dairy products, while young Japanese women who were obese had an increased intake of unhealthy foods, such as confectioneries. Therefore, the potential for unhealthy eating habits is likely to be present in all young Japanese women, but measures may need to be adapted according to body size.

## Figures and Tables

**Table 1:** Characteristics of Japanese women aged 20–39 years in the 2001–2019 National Health and Nutrition Survey.

Year		2001			2002		2003			2004				2005		2006		2007			2008		2009			2010	
*n*	All	1133			938		913			736				630		773		729			664		657			595	
	Underweight	202			185		163			131				127		125		137			122		119			119	
	Normal	796			664		647			550				431		565		524			472		465			405	
	Obese	135			89		103			55				72		83		68			70		73			71	
		Mean		SE	Mean	SE	Mean		SE	Mean		SE		Mean	SE	Mean	SE	Mean	SE		Mean	SE	Mean	SE		Mean	SE
Age (years)	Underweight	29.7		0.4	28.3	0.4	29.3		0.5	29.5		0.5		30.2	0.5	29.5	0.5	29.8	0.5		30.3	0.6	29.6	0.6		29.1	0.5
	Normal	30.5		0.4	30.8	0.4	30.9		0.5	30.8		0.5		30.6	0.5	31.1	0.5	31.7	0.5		31.2	0.6	31.3	0.6		31.9	0.5
																					
	Obese	32.3		0.5	32.8	0.6	32.3		0.5	32.1		0.7		32.8	0.6	32.3	0.5	32.5	0.6		32.3	0.6	33.5	0.5		33.5	0.6
Body mass index (kg/m^2^)	Underweight	17.5		0.1	17.5	0.1	17.7		0.1	17.5		0.1		17.5	0.1	17.6	0.1	17.6	0.1		17.5	0.1	17.6	0.1		17.5	0.1
	Normal	20.9		0.1	20.8	0.1	20.9		0.1	20.8		0.1		20.9	0.1	20.9	0.1	20.8	0.1		20.9	0.1	20.8	0.1		21.1	0.1
	Obese	27.9		0.2	28.2	0.3	28.5		0.3	27.8		0.3		28.6	0.4	28.8	0.4	27.7	0.3		29.1	0.5	28.8	0.5		27.9	0.3
Step count (/day)	Underweight	7681		248	7952	270	7379		293	7065		332		7095	328	7757	352	6762	291		6709	321	7551	298		6998	324
	Normal	7910		134	7952	146	7633		137	7065		158		7286	170	7753	161	6936	159		6933	153	7487	172		6940	174
	Obese	6922		291	6459	292	6500		308	6950		453		6962	428	7179	404	6934	482		5922	373	7760	410		6787	373
Year		2011		2012		2013			2014			2015		2016		2017		2018			2019		Lower endpoint		Upper endpoint	APC (%)	*p*-value
*n*	All	575		1870		503			440			377		1310		299		362			267						
	Underweight	101		346		95			70			73		244		49		74			46						
	Normal	410		1301		342			306			273		886		217		245			186						
	Obese	64		223		66			64			31		180		33		43			35						
		Mean	SE	Mean	SE	Mean		SE	Mean		SE	Mean	SE	Mean	SE	Mean	SE	Mean		SE	Mean	SE					
Age (years)	Underweight	30.2	0.6	31.1	0.3	30.7		0.6	30.1		0.8	30.1	0.7	30.7	0.4	29.4	0.9	31.8		0.6	30.0	0.9				0.4	0.001
	Normal	31.5	0.6	31.8	0.3	31.4		0.6	30.7		0.8	31.2	0.7	31.6	0.4	31.2	0.9	30.7		0.6	30.9	0.9	2001		2011	0.4	0.001
																							2011		2019	−0.3	0.066
	Obese	32.3	0.8	33.0	0.3	32.3		0.7	31.5		0.7	30.8	1.0	32.9	0.4	32.9	0.8	32.1		1.0	32.0	0.8				0	-
Body mass index (kg/m^2^)	Underweight	17.6	0.1	17.6	0.0	17.5		0.1	17.5		0.1	17.6	0.1	17.6	0.1	17.5	0.1	17.6		0.1	17.7	0.1				0	-
	Normal	21.0	0.1	21.0	0.1	20.9		0.1	21.0		0.1	20.8	0.1	20.9	0.1	21.0	0.1	21.1		0.1	21.2	0.1				0	-
	Obese	28.2	0.3	29.0	0.2	28.5		0.4	29.0		0.4	27.8	0.6	28.7	0.2	29.1	0.8	28.2		0.5	28.3	0.4				0.1	0.063
Step count (/day)	Underweight	7090	356	6718	185	7161		350	6807		443	7324	447	6859	285	6179	459	6631		341	5554	512				−0.9	<0.001
	Normal	7505	189	7037	107	7453		219	7247		230	7495	230	7213	131	7540	266	6831		228	7198	294				−0.5	0.013
	Obese	7195	496	7329	270	6337		455	5992		411	5787	662	6638	245	5761	602	6909		568	7071	751				−0.1	0.823

APC, annual percentage change. The *p*-value was analysed by using Joinpoint regression analysis.

## Data Availability

Data cannot be shared because the Ministry of Health, Labour, and Welfare permits individuals who applied for secondary use of the NHNS data to refer to the data.

## Supplementary Materials

The following supporting information can be downloaded at: https://www.mdpi.com/article/10.3390/nu14194078/s1, Table S1: Definitions of food groups in this study; Table S2: Trends in food group intakes among young Japanese women classified as underweight, normal and obese between 2001 and 2019.
